# Understanding the interactions between iron supplementation, infectious disease and adverse birth outcomes is essential to guide public health recommendations

**DOI:** 10.1186/s12916-019-1376-8

**Published:** 2019-08-05

**Authors:** Freya J. I. Fowkes, Eliza Davidson, Paul A. Agius, James G. Beeson

**Affiliations:** 1 Maternal and Child Health Program, Burnet Institute, Melbourne, VIC 3004 Australia; 2 Department of Epidemiology and Preventive Medicine, Monash University, Melbourne, VIC 3004 Australia; 3 Melbourne School of Population and Global Health, The University of Melbourne, Melbourne, VIC 3010 Australia; 4 Department of Infectious Diseases, Central Clinical School, Monash University, Melbourne, VIC 3004 Australia; 5 Department of Medicine (RMH), The University of Melbourne, Melbourne, VIC 3010 Australia

**Keywords:** Iron deficiency, Low birth weight, Preterm birth, Malaria, Anaemia, Pregnancy

## Abstract

Pregnant women are highly susceptible to anaemia and iron deficiency due to the increased demands of pregnancy as well as other factors. Iron supplementation is recommended in pregnancy, yet the benefits on newborn outcomes are variable between populations, most likely due to the heterogeneity in the prevalence of iron deficiency, detrimental birth outcomes and infectious diseases. Furthermore, there are concerns regarding iron supplementation in malaria-endemic areas due to reports of increased risk of malaria in those receiving iron. This is compounded by limited knowledge of how iron deficiency, anaemia, malaria, and other infections may interact to influence birth outcomes. In a recent cohort study in Papua New Guinea, where there is a high burden of infections and iron deficiency, we found that iron deficiency in pregnancy was associated with a reduced risk of adverse birth outcomes. However, this effect could not be wholly explained by interactions between iron deficiency and malaria. We proposed that iron deficiency may confer a degree of protection against other infectious pathogens, which in turn caused improvements in birthweight. We argue that further studies in multiple populations are crucial to elucidate interactions between iron status, iron supplementation and birthweight as well as to understand the context-specific benefits of iron supplementation in pregnancy and inform public policy. Focus should be given to haematological studies on anaemia, haemodilution and iron absorption, as well as investigating infectious diseases and other nutritional deficiencies. This is a particular priority in resource-constrained settings where the prevalence of iron deficiency, poor nutrition, infections and poor birth outcomes are high. While current recommendations of iron supplementation and malaria prophylaxis to reduce the burden of poor pregnancy outcomes should be supported, the strength of evidence underpinning these must be improved and new insights should be garnered in order to maximise improvements in maternal and child health.

Please see related article: https://bmcmedicine.biomedcentral.com/articles/10.1186/s12916-018-1146-z.

Please see related article: https://bmcmedicine.biomedcentral.com/articles/10.1186/s12916-019-1375-9.

## Background

Understanding the complex interactions between nutritional deficiencies and infectious diseases is vital for informing key policy and treatment guidelines in resource-poor settings to ensure gains in maternal and child health. Pregnant women are highly susceptible to anaemia and iron deficiency due to the increased iron demands of pregnancy in conjunction with other factors [1]. To prevent these conditions from developing and to improve newborn and infant outcomes, the World Health Organization (WHO) currently recommends universal iron supplementation throughout pregnancy together with “measures to prevent, diagnose and treat malaria” in malaria-endemic areas [2]. However, there are concerns regarding the value of antenatal iron supplementation in malaria-endemic areas due to reports of increased risk of malaria in those receiving iron supplementation [3, 4]. These issues are compounded by a lack of understanding of how iron deficiency, anaemia and malaria may interact to influence birth outcomes. Additionally, published reports indicate variable effects of iron supplementation in pregnancy on newborn outcomes [5]. In our recent article published in *BMC Medicine* [6], we investigated iron deficiency and pregnancy outcomes in a cohort of Papua New Guinean women and found that iron deficiency in pregnancy was associated with a reduced risk of adverse birth outcomes (low birthweight and premature delivery); this association was not simply explained by a potential protective effect of iron deficiency against malaria. We hypothesised that an interaction between iron deficiency and other infectious diseases may explain the observed effect or that other nutritional factors may be contributing to these associations. Furthermore, it is essential to understand the mechanism(s) underlying these interactions in resource-limited settings where the burden of infectious diseases and poor nutrition are high. While our findings raise questions about the benefits of iron supplementation in these settings and shed light on potential causal pathways, they do not constitute sufficient evidence to recommend that iron supplementation be stopped in pregnancy. Furthermore, there are other benefits of iron supplementation for the mother and infant that need to be considered in policy decisions. Instead, we concluded that it is essential to provide both iron supplementation for anaemia and effective malaria prophylaxis during pregnancy according to current WHO policy [6], integrated with interventions that address other nutritional deficiencies or infections that may potentially achieve greater health benefits.

## The benefits of antenatal iron supplementation on birthweight appear to be context specific

The current WHO global recommendations are based on findings from a Cochrane systematic review of daily iron supplementation trials in pregnancy that found an overall benefit of iron supplementation, although the effects on different outcomes were variable between populations [5]. Verhoef et al. [7] note that our findings potentially contradict previous trial evidence that iron supplementation leads to increased birthweight. While observational cohort studies of iron deficiency are not equivalent to randomised controlled trials of iron supplementation, it is important to consider all the evidence given the quality and heterogeneous results of iron supplementation trials. While the Cochrane review found extensive evidence that iron supplements improved maternal haemoglobin levels and risk of iron deficiency [5], few studies examined the relationship between iron supplementation and birthweight. Further, the available evidence was deemed ‘not of high quality’ and the results uncertain [5]. From 15 trials (the vast majority of studies recruited iron-replete, non-anaemic women), the meta-analysis found that iron-supplemented women had infants with modestly increased birthweight compared to those who did not receive iron (median difference in birthweight of 23.75 g, 95% CI − 3.02 to 50.51 g; thus, daily iron supplementation decreased or increased birthweight by as much as 3 g or 50 g, respectively) [5]. Furthermore, the magnitude of the effect that iron supplementation had on birthweight varied substantially between studies. Only two studies (one in the USA [8] and one in Nepal [9]) showed a statistically significant effect of iron supplementation in their own right. Of note, most studies observed no significant birthweight differences for supplemented versus control groups [5]. The only iron supplementation randomised controlled trial to include iron-deficient women (published after the Cochrane review), set in a malaria-endemic part of Kenya and undertaken by the authors of the corresponding letter (Mwangi et al. [10]), found an overall mean birthweight increase of 150 g (95% CI 56–244 g) in supplemented women compared to placebos. However, it is unclear if this magnitude of effect observed in a single study extrapolates to other resource-poor settings. In Papua New Guinea, the setting of our study, very few studies have assessed the effects of iron deficiency or supplementation, or both, on pregnancy outcomes. An early cohort study by Oppenheimer et al. [11] examined the impact of total dose intravenous iron infusion given to women with severe anaemia on infant outcomes, finding no evidence of an effect on birthweight. Given the heterogeneity in the magnitude of effect of iron supplementation on birthweight across different settings, our findings may not be entirely contradictory of published data. This raises the question of whether the benefits of iron supplementation are specific to certain populations or settings. It is difficult to quantify the impact of iron supplementation in resource-poor settings with different setting-specific risks of low birthweight (i.e. where the prevalence of iron deficiency, malaria and other infectious diseases, as well as other nutritional deficiencies, is high). The current evidence, as it stands, supports the current WHO guidelines, but there is clearly a need for studies to understand the heterogeneous findings to provide further context-specific policies to maximise health impacts.

## Mechanisms underpinning the association between iron deficiency or supplementation and birthweight

Verhoef et al. propose that one possible explanation for the findings of a protective effect of iron deficiency on adverse birth outcomes in our study is that women who were iron deficient at enrolment may have absorbed more iron from supplements, or benefitted more from supplementation, than their iron-replete counterparts, resulting in higher birthweight among those who tested iron deficient at first antenatal visit. They seek to support this hypothesis by reanalysing data from their previously published iron supplementation trial conducted in Kenya [10]. There are caveats to consider in post hoc exploratory sub-analyses, and the overall effect described had modest statistical and clinical significance. In terms of clinical significance, the authors do not quantify the average moderating effect of iron-level on the association between iron supplementation and birthweight. Reading the right panel of the figure, we estimate a moderating effect of 50 g mean reduction in effect per unit increase in body iron index (and calculate a 95% CI of 37–62 g, assuming *p* = 0.04 and a *t*-distribution). Given this, the impact of iron deficiency on modifying the influence of supplementation on birthweight may in fact be small and may not fully explain results in other populations such as ours where we observed a mean difference in birthweight between iron-deficient and iron-replete women of 230 g (95% CI 118, 514). Interpretation of the generalisability of their findings is hindered by the lack of definition as to what constitutes discrete iron-depletion risk and little reference to the ‘iron status’ sample distribution in their study population making it hard to extrapolate their finding to other study populations with different distributions of iron status, inflammation and malaria.

We are unable to fully explore the iron absorption hypothesis proposed by Verhoef et al. in our study. While all women in the study were prescribed daily iron and folate supplementation throughout pregnancy from enrolment, we do not have data on supplement accessibility or compliance (although one would assume that, in the absence of knowing their iron status, iron supplementation uptake would be similar in both iron-replete and iron-deficient women). However, we did investigate, through causal mediation analysis, whether the protective effect of iron deficiency was mediated through anaemia (both at enrolment and at prospective time points throughout pregnancy), which could serve as a proxy for levels of iron absorption [6]. Assuming that iron supplementation does improve haemoglobin concentration differentially in iron-deficient and iron-replete women, thus improving birthweight, we would expect to find a significant proportion of the protective effect to be mediated through that pathway. Instead, we found that only a small proportion (< 10%) of the protective association between iron deficiency and birthweight was mediated through anaemia [6]. Nevertheless, this finding, with its caveats (e.g. measurement error of iron absorption through an anaemia proxy), does not fully discount the hypothesis of Verhoef et al.; instead, it highlights the need for further research into the underlying haematological mechanisms of iron supplementation or deficiency, or both, and the risk of adverse birth outcomes, especially in settings with a high burden of nutrition deficiencies and infections.

An alternative hypothesis we put forward in our recent article is that iron deficiency may confer a degree of protection against infections, which in turn results in improved birthweight as observed in our study [6]. Iron is an essential nutrient for the survival and growth of a number of pathogens [10]; thus, host iron deficiency reduces pathogen fitness, decreasing infection susceptibility or severity, or both [12, 13]. In our study, only a small proportion of the association between iron deficiency and birthweight was mediated through malaria and anaemia, while a large proportion of the protective effect was independent of these aetiologies, suggesting that risk factors other than malaria and anaemia determine low birthweight in this population. Pregnant women in Papua New Guinea are exposed to a range of viral and bacterial pathogens and parasitic infections, and therefore, a reduction of infections resulting from iron deficiency may explain the improved birth outcomes in iron-deficient women. Supporting this theory, the effect of iron deficiency on birthweight in our study was the greatest in primigravidae women, who are more susceptible to, and more severely affected by, infection [14]. Elucidating the mechanistic interactions between iron deficiency, infectious disease and birth outcomes is also warranted to inform guidelines, particularly across multiple populations with a varying burden of these global public health problems.

## Implications for programmes and policies

We argue that further studies are crucial to elucidate the interactions between iron status, iron supplementation and birthweight in order to understand the context-specific benefits of iron supplementation in pregnancy and to inform public policy. Detailed haematological studies, as well as those investigating infectious diseases and other nutritional deficiencies, are needed, particularly in resource-constrained settings where the prevalence of iron deficiency, poor nutrition, infections and poor birth outcomes is high. Further studies in multiple populations to understand the generalisability of associations between iron deficiency and birthweight are also needed, as well as studies to determine the mechanisms and causal pathways underlying the relationships between iron status and pregnancy outcomes. In the absence of an in-depth understanding of these interactions, the WHO recommendation that both iron supplementation and malaria prophylaxis are provided to reduce the burden of poor pregnancy outcomes should be supported; clearly, however, the strength of evidence to support this needs to be improved—these new insights may enable great improvements in maternal and child health.

## Data Availability

Not applicable.
